# Pre-existing helminth infection impairs the efficacy of adjuvanted influenza vaccination in mice

**DOI:** 10.1371/journal.pone.0266456

**Published:** 2022-03-31

**Authors:** Wiebke Hartmann, Marie-Luise Brunn, Nadine Stetter, Gülsah Gabriel, Minka Breloer

**Affiliations:** 1 Section for Molecular Biology and Immunology, Helminth Immunology Group, Bernhard Nocht Institute for Tropical Medicine, Hamburg, Germany; 2 Research Department for Viral Zoonoses—One Health, Leibniz Institute for Experimental Virology, Hamburg, Germany; 3 Institute of Virology, University for Veterinary Medicine, Hanover, Germany; 4 Department for Biology, University Hamburg, Hamburg, Germany; IRNASA: Instituto de Recursos Naturales y Agrobiologia de Salamanca, SPAIN

## Abstract

The world health organization estimates that more than a quarter of the human population is infected with parasitic worms that are called helminths. Many helminths suppress the immune system of their hosts to prolong their survival. This helminth-induced immunosuppression “spills over” to unrelated antigens and can suppress the immune response to vaccination against other pathogens. Indeed, several human studies have reported a negative correlation between helminth infections and responses to vaccinations. Using mice that are infected with the parasitic nematode *Litomosoides sigmodontis* as a model for chronic human filarial infections, we reported previously that concurrent helminth infection impaired the vaccination-induced protection against the human pathogenic 2009 pandemic H1N1 influenza A virus (2009 pH1N1). Vaccinated, helminth-infected mice produced less neutralizing, influenza-specific antibodies than vaccinated naïve control mice. Consequently helminth-infected and vaccinated mice were not protected against a challenge infection with influenza virus but displayed high virus burden in the lung and a transient weight loss. In the current study we tried to improve the vaccination efficacy using vaccines that are licensed for humans. We either introduced a prime-boost vaccination regimen using the non-adjuvanted anti-influenza vaccine Begripal or employed the adjuvanted influenza vaccine Fluad. Although both strategies elevated the production of influenza-specific antibodies and protected mice from the transient weight loss that is caused by an influenza challenge infection, sterile immunity was not achieved. Helminth-infected vaccinated mice still had high virus burden in the lung while non-helminth-infected vaccinated mice rapidly cleared the virus. In summary we demonstrate that basic improvements of influenza vaccination regimen are not sufficient to confer sterile immunity on the background of helminth-induced immunosuppression, despite amelioration of pathology i.e. weight loss. Our findings highlight the risk of failed vaccinations in helminth-endemic areas, especially in light of the ongoing vaccination campaign to control the COVID-19 pandemic.

## Introduction

Vaccination is the most successful prophylactic intervention against life-threatening infectious diseases. However, there are numerous factors that dramatically influence vaccine-induced immunogenicity, like pre-existing infections. Helminth parasites infect more than a quarter of the human population [1, 2] and inflict significant changes to the immunological status of their hosts [3–5]. Several studies report a negative correlation between pre-existing helminth infections and the response to vaccinations against cholera [6], tuberculosis [7], tetanus [8], influenza [9], or malaria [10], in the human population; a fact that is seldom taken into account during testing of novel vaccines in the tropics [11, 12]. Especially in light of the global efforts to control the coronavirus disease 19 (COVID-19) pandemic by vaccination, a negative impact of concurrent helminth infection of vaccination efficacy should be addressed [13, 14].

To investigate possible interference with vaccination efficacy by underlying helminth infections in a controlled setting, we use *Litomosoides sigmodontis*-infected mice as a model for chronic human filarial infections [15]. *L*. *sigmodontis* infective third stage larvae (L3) are transmitted by mites, the intermediate host, during the blood meal. Although the natural definitive hosts are cotton rats (*Sigmodon hispidus*), exposure of inbred laboratory mice to infected mites results in successful infection. The mites are separated from the mice after the blood meal and transmitted L3 migrate within 4 days via the lymphatics to the thoracic cavity of the mice. They moult via fourth stage larvae (L4) to parasitic adults by day 30 post infection (p.i.). Semi-susceptible C57BL/6 mice allow establishment of infection but start to kill the parasites by day 35 via granuloma formation while fully susceptible BALB/c mice stay infected for more than 90 days [16, 17]. Using this model, we reported previously that acute and chronic *L*. *sigmodontis* infection reduced antigen-specific T cell proliferation and antibody (Ab) responses of all isotypes to model antigen immunization in both, BALB/c and C57BL/6 mice [18–21]. These findings were clinically relevant: First, the expansion of sporozoite-specific CD8^+^ T cells, their cytokine production, cytotoxic activity and protection against a *Plasmodium berghei* challenge infection was impaired in vaccinated helminth-infected mice compared to non-infected mice [22]. Second, vaccination responses to the trivalent split subunit vaccine Begripal, that was licensed for human influenza vaccination in the seasons 2014 to 2017, were impaired by concurrent helminth infection [23]. We recorded reduced quantity of all isotypes and impaired quality of Influenza Haemagglutinin (HA)-specific Ab response in helminth-infected mice compared to naïve control mice. Vaccinated control mice were protected against a challenge infection with the human pathogenic 2009 pandemic H1N1 influenza A virus (2009 pH1N1) while vaccinated, helminth-infected mice displayed transient weight loss and high virus burden in the lung. Moreover, reduced Ab responses were also observed if vaccinations were performed after immune-driven or drug-induced termination of the *L*. *sigmodontis* infection [23, 24]. This sustained suppression of vaccine response was correlated to a systemic and sustained expansion of type 1 regulatory T cells (Tr1) and could be partially reverted by blocking the Tr1 key cytokine IL-10 [21, 23, 24]. In summary, our findings raise the concern that also humans living in helminth-endemic areas that either suffer from acute helminth infection or have a history of previous helminth infections may not benefit from vaccinations even the absence of acute and thus diagnosable helminthiasis.

Here we use the *L*. *sigmodontis* mouse model to test improved vaccination strategies that would confer protection on the background of pre-existing helminth infection. Next to prime-boost vaccinations, the addition of “enhancers” that are called adjuvants is a common strategy to increase the immunogenicity of vaccinations [25]. While the trivalent split subunit influenza vaccine Begripal is delivered without adjuvants, the influenza vaccine Fluad is enhanced with MF59, an oil-in-water squalene-based adjuvant that is licensed for humans [25]. We show that either prime-boost vaccination with the non-adjuvanted influenza vaccine Begripal or single vaccination with the adjuvanted influenza vaccine Fluad did not abrogate the helminth-induced reduction of the Ab response. Adjuvanted or prime-boost vaccination rather elevated the Ab response in general, in helminth-infected and naïve control mice to the same extent. This elevated Ab response in vaccinated and helminth-infected mice was sufficient to protect against the transient weight loss induced by influenza virus challenge infection. However, the improved vaccination regimen was not sufficient to prevent viral replication, as influenza virus load in the lungs were still significantly higher in vaccinated, helminth-infected mice compared to mice that were vaccinated in the absence of an underlying helminth infection.

## Materials and methods

### Ethics and mice

All animal experimentations were conducted at the specific pathogen-free animal facility of the Bernhard Nocht Institute for Tropical Medicine (BNITM) in agreement with the German Animal Welfare Act and the relevant German authority (Behörde für Gesundheit und Verbraucherschutz, Hamburg, approval numbers 84/15, 103/2018. All mice were kept in individually ventilated cages. C57BL/6 mice were either bred in the animal facility of the BNITM or were obtained from Janvier Labs. Female 8–10 week old mice were used for experiments. To alleviate suffering, all mice are kept in small groups of 2–5 with toys and crawl spaces to allow hiding places. Experimental mice were monitored daily and scored for signs of suffering (ruffled fur, impaired activity, signs of paralysis) and body weight. Mice losing more than 25% of their body weight or reaching other endpoint criteria according to the score were sacrificed. For anaesthesia, mice received ketamine/xylazine (100 mg and 5 mg/kg body weight) i.p. in 200 μl PBS and were closely monitored and kept on a heated mat (37°C) until they regained consciousness. Mice were sacrificed by an overdosed CO_2_ Narcosis. When the inter-toe reflex was no longer apparent a cervical dislocation was performed in addition.

### *L*. *sigmodontis* and influenza virus infection

The life cycle of *L*. *sigmodontis* was maintained in their natural reservoir, the cotton rats (*Sigmodon hispidus*). Therefore, cotton rats were anesthetized and blood was collected from the retro-bulbar sinus in order to count the microfilariae (MF, L1). Cotton rats, which were used for further infection of blood-sucking mites (*Ornithonyssus bacoti*), had an infection rate of 500–2000 MF per μl blood. Infected cotton rates were exposed to mites that ingested MF during a blood meal. Infected mites were kept at 29°C and 90% humidity for 14 days to allow maturation of L1 to L3. Experimental mice were anesthetized and exposed to these infected mites for 16 hours, i.e. naturally infected.

For influenza infection C57BL/6 mice were anesthetized and i.n. infected with 25 μL 1 x 10^3^ plaque forming units (PFU) 2009 pH1N1 influenza A/Hamburg/05/09 virus. The influenza virus was isolated from pharyngeal swabs of a female patient as described previously [26]. Health status of the mice was monitored daily according to the animal protocols approved by the Hamburg authorities. Body weight was measured at the indicated time points of infection either until non-vaccinated mice regained their original body weight or for 14 days.

### Vaccination and quantification of the vaccine-specific humoral response

Mice were vaccinated by i.p. injection of 3.75 μg either non-adjuvanted (Begripal, Seqirus) or adjuvanted (Fluad, Seqirus) vaccine against influenza in 200 μL PBS. Blood was collected from the vena fascialis at the indicated time points, 2–4 weeks after vaccination and allowed to coagulate for 1h at RT. After centrifugation (10.000 x g for 10 min) serum was transferred into a fresh tube and stored at -20°C until further analysis.

### Quantification of HA-specific IgG1

ELISA High Binding Microlon (Greiner) plates were coated overnight with 1 μg/mL Begripal. Plates were washed and blocked for 2 h with 100 μL 1% BSA in PBS. After 2 h incubation with serially diluted serum in duplicates, plates were washed and incubated for 1 h with HRP-labeled anti-mouse IgG1 (Invitrogen). After a further washing step, plates were developed with 100 μL tetramethylbenzidine (0.6 mg/mL in DMSO), 0.003% H_2_O_2_ in 100 mM NaH_2_PO_4_ (pH 5.5) for 2.5 min. Reaction was stopped by addition of 25 μL 2 M H_2_SO_4_ per well and OD_450_ was measured. The titre is defined as the last serum dilution that results in an OD_450_ above the double background.

### Hemagglutinin inhibition (HI) assay

Murine serum samples were thawed and heat inactivated for 30 min at 56°C in a water bath. Serial dilutions of sera in 25 μL PBS were incubated with 25 μl of 2009 pH1N1 influenza A virus in duplicates in 96-well-V-plates. After 30 min incubation at room temperature, 50 μL 1% fresh chicken erythrocytes solution in 0.9% NaCl was added to each well. The serum dilution that still inhibited agglutination was calculated as a titre after a further 1-hour-incubation at 4°C. Prior to the HI assay the virus solution was standardized to 8 HI units. To this end the hemagglutination capacity of the of 2009 pH1N1 influenza A virus was determined by serial dilutions of the virus solution and incubation with chicken erythrocytes solution as described above. The minimal amount of virus that still caused complete agglutination of red blood cells is defined as HI unit of 1.

### Detection of viral loads in the lungs

Viral loads were determined in lung homogenates by MDCK plaque assay. Lungs were removed at indicated time points and stored at -70°C in 0.1% BSA in PBS. Lungs were thawed and homogenized. The supernatant was collected after centrifugation (950 x g, 10 min, 4°C). Serial dilutions of lung homogenates (10^1^ to 10^5^) were added to a confluent MDCK cell culture in 6-well-plates. After 30 min at 37°C, plates were overlaid with 3 mL 1.25% Avicel in MEM containing 1μg/mL tolylsulfonyl phenylalanyl chloromethyl ketone (TPCK)-Trypsin. After a further incubation for 72 h at 37°C and 5% CO_2_, the overlay was removed and the plates were washed with PBS. The cells were fixed with 0.5 mL 4% paraformaldehyde (PFA) for 30 min at 4°C. PFA was removed and plates were incubated for 10 min with 1 mL/well 1% crystal violet. The staining was stopped by removing crystal violet and washing the plates with tap water.

### Statistical analysis

**Fig 1:**
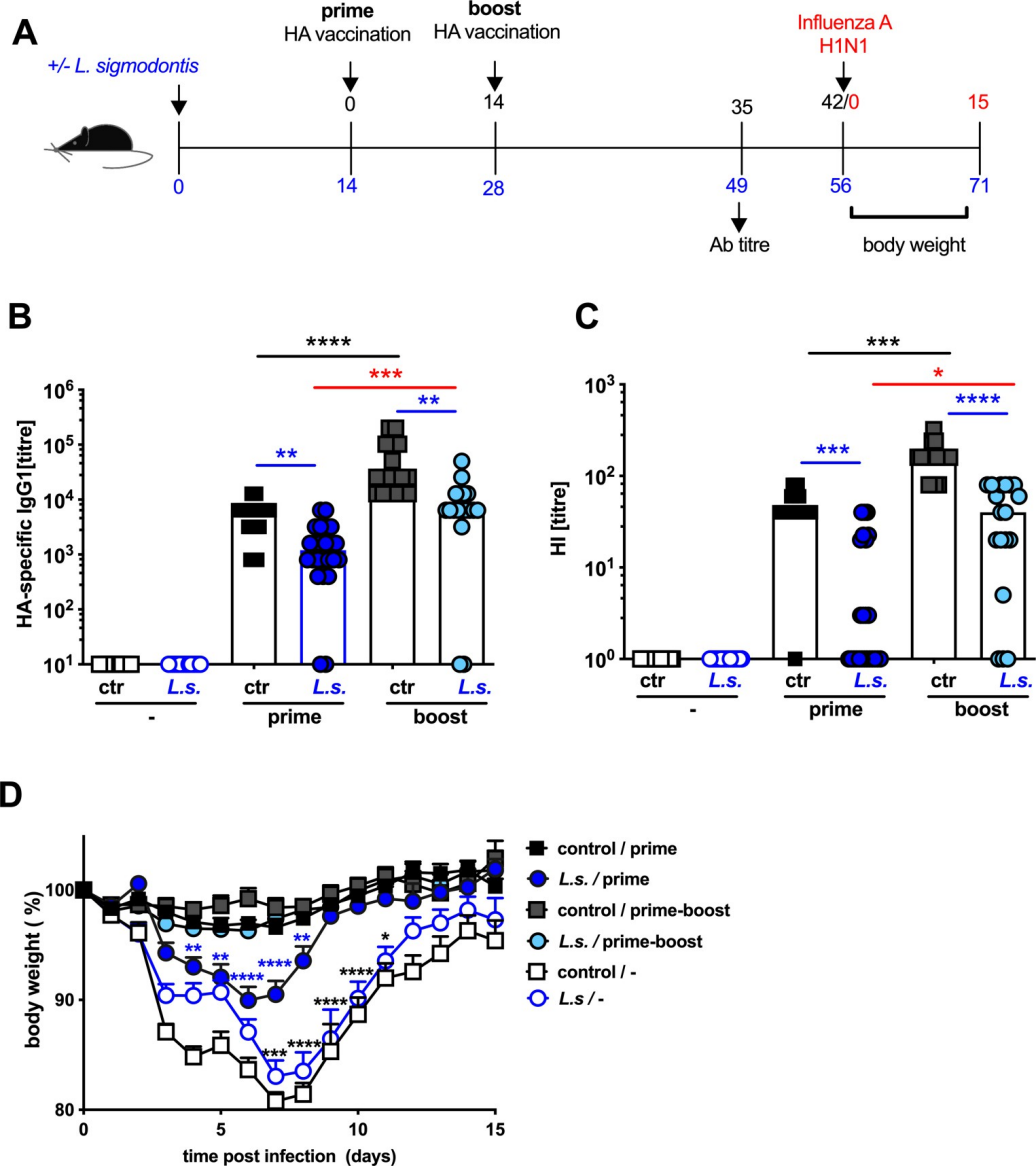
Prime-boost influenza vaccination protects *L*. *sigmodontis*-infected mice from challenge infection-induced weight loss. **(A)** Experimental design: C57BL/6 mice were left naïve (black and grey squares) or naturally infected with *L*. *sigmodontis* (light and dark blue circles). Mice either received a control injection (open symbols) or were i.p. vaccinated with non-adjuvanted influenza vaccine Begripal season 2017/18 (black squares, dark blue circles) either once at day 14 post *L*. *sigmodontis* infection or in a prime-boost regimen (grey squares, light blue circles) at day 14 and day 28 post *L*. *sigmodontis* infection. **(B)** Titres of HA-specific IgG1 and **(C)** HI titres were quantified 21 days after the last vaccination. **(D)** All mice were i.n. infected with 1 x 10^3^ PFU 2009 pH1N1 influenza A 1 week later and body weight was recorded daily over 15 days. **(BC)** show combined results of 3 independent experiments with n ≥ 6 per group. Each symbol represents an individual mouse, the bars show the median. **(D)** shows combined results out 3 independent experiments with n ≥ 4 per group and experiment. Symbols show the mean per group at the indicated time point, error bars show SD. Asterisks indicate statistically significant differences applying **(BC)** Kruskal-Wallis test (p > 0.001) with Dunn’s multiple comparison test comparing all vaccinated groups with each other (* *p* ≤ 0.05, ** *p* ≤ 0.01, *** *p* ≤ 0.001, **** *p* ≤ 0.0001; black asterisks: naïve control single-vaccinated group to naïve control prime-boost-vaccinated group, blue asterisks: *L*. *sigmodontis*-infected single- or prime-boost-vaccinated groups to naïve control single- or prime-boost-vaccinated groups, red asterisks: *L*. *sigmodontis*-infected single-vaccinated to *L*. *sigmodontis*-infected prime-boost vaccinated groups. **(D)** shows 2-way ANOVA with Sidak`s multiple comparison test comparing the control single-vaccinated group to *L*. *sigmodontis*-infected, single-vaccinated group (blue asterisks) and the *L*. *sigmodontis*-infected single-vaccinated group to *L*. *sigmodontis*-infected non-vaccinated group (black asterisks).

**Fig 2:**
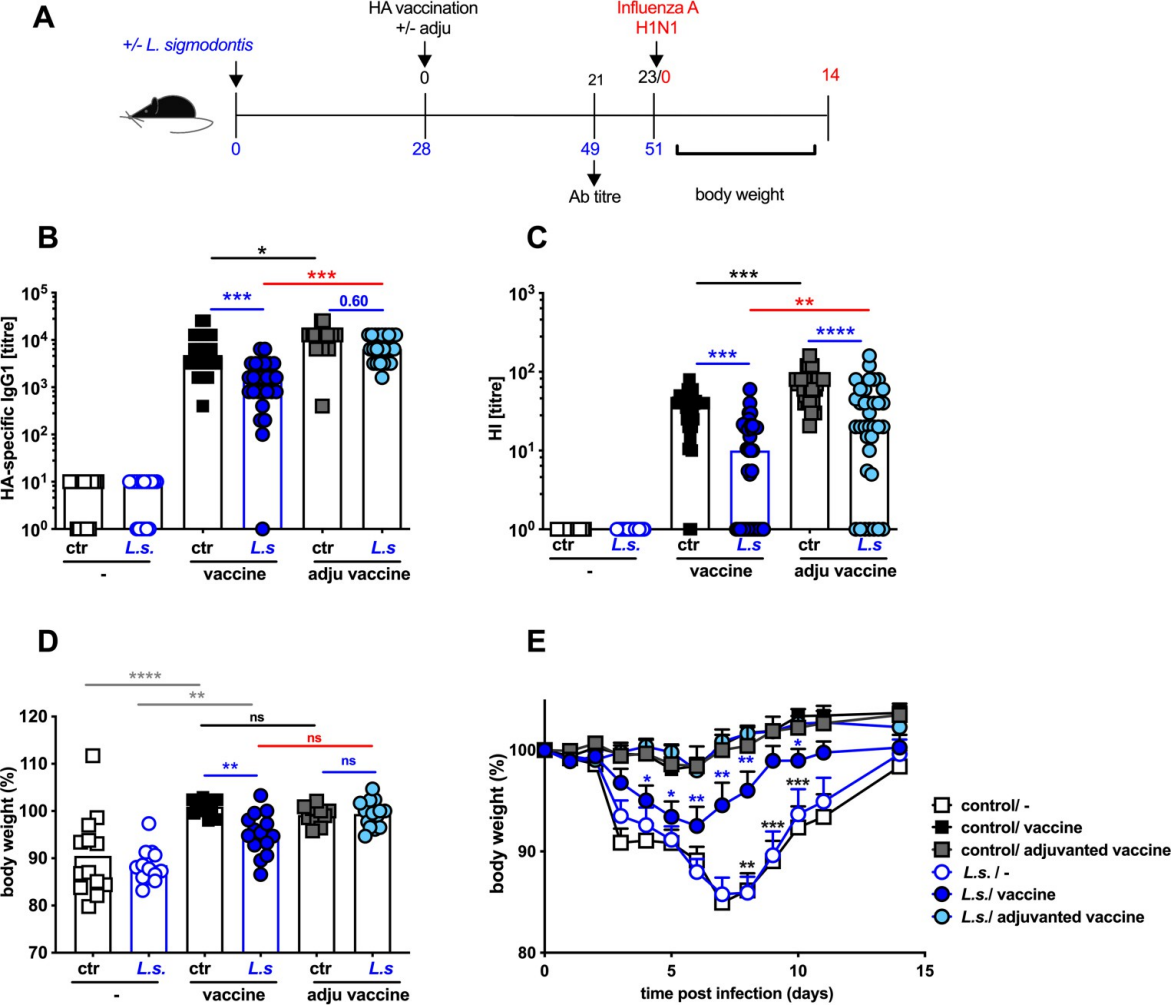
Adjuvanted influenza vaccination protects *L*. *sigmodontis*-infected mice from challenge infection-induced weight loss. **(A)** Experimental design: C57BL/6 mice were left naive (black and grey squares) or naturally infected with *L*. *sigmodontis* (light and dark blue circles). Mice either received a control injection (open symbols) or were i.p. vaccinated with non-adjuvanted influenza vaccine Begripal season 2017/18 (black squares, dark blue circles) or adjuvanted influenza vaccine Fluad season 2017/18 (grey squares, light blue circles) in 200 μl at day 28 post *L*. *sigmodontis* infection. **(B)** Titres of HA-specific IgG1 and **(C)** HI titres were quantified 21 days after vaccination. **(D-G)** All mice were i.n. infected with 1 x 10^3^ PFU 2009 pH1N1 influenza A 2 days later. Body weight was recorded day 3 post challenge infection **(D)** or at indicated time points **(E).** Shown are combined results out of 4 **(BC)** or 2 **(DE)** independent experiments with n ≥ 5 **(B-D)** or n ≥ 4 **(E)** per group and experiment. **(BC)** Each symbol represents an individual mouse the lines show the median. Asterisks indicate statistically significant differences applying Kruskal-Wallis test (p > 0.001) with Dunn’s multiple comparison test **(BC)** comparing all vaccinated groups with each other. Numbers indicate p-values, ns = not significant, * *p* ≤ 0.05, ** *p* ≤ 0.01, *** *p* ≤ 0.001, **** *p* ≤ 0.0001; black asterisks: naïve control non-adjuvanted vaccine group to naïve control adjuvanted vaccine group, blue asterisks: *L*. *sigmodontis*-infected non-adjuvanted or adjuvanted vaccine groups to naïve control non-adjuvanted or adjuvanted vaccine groups, red asterisks: *L*. *sigmodontis*-infected non-adjuvanted to *L*. *sigmodontis*-infected adjuvanted vaccine groups. **(D)** Each symbol represents an individual mouse, the lines show the mean, asterisks indicate statistically significant differences applying 1-way ANOVA (p > 0.001) with Tukey’s multiple comparison test comparing all groups with each other, colours as indicated above and grey asterisks: non-vaccinated to vaccinated groups. **(E)** Symbols show the mean per group at the indicated time point, error bars show SEM and asterisks show difference between the control adjuvanted vaccine group to *L*. *sigmodontis*-infected adjuvanted vaccine group (blue asterisks) or differences between the *L*. *sigmodontis*-infected non-adjuvanted vaccine group to *L*. *sigmodontis*-infected non-vaccinated group (black asterisks) applying 2-way ANOVA with Sidak`s multiple comparison test.

**Fig 3:**
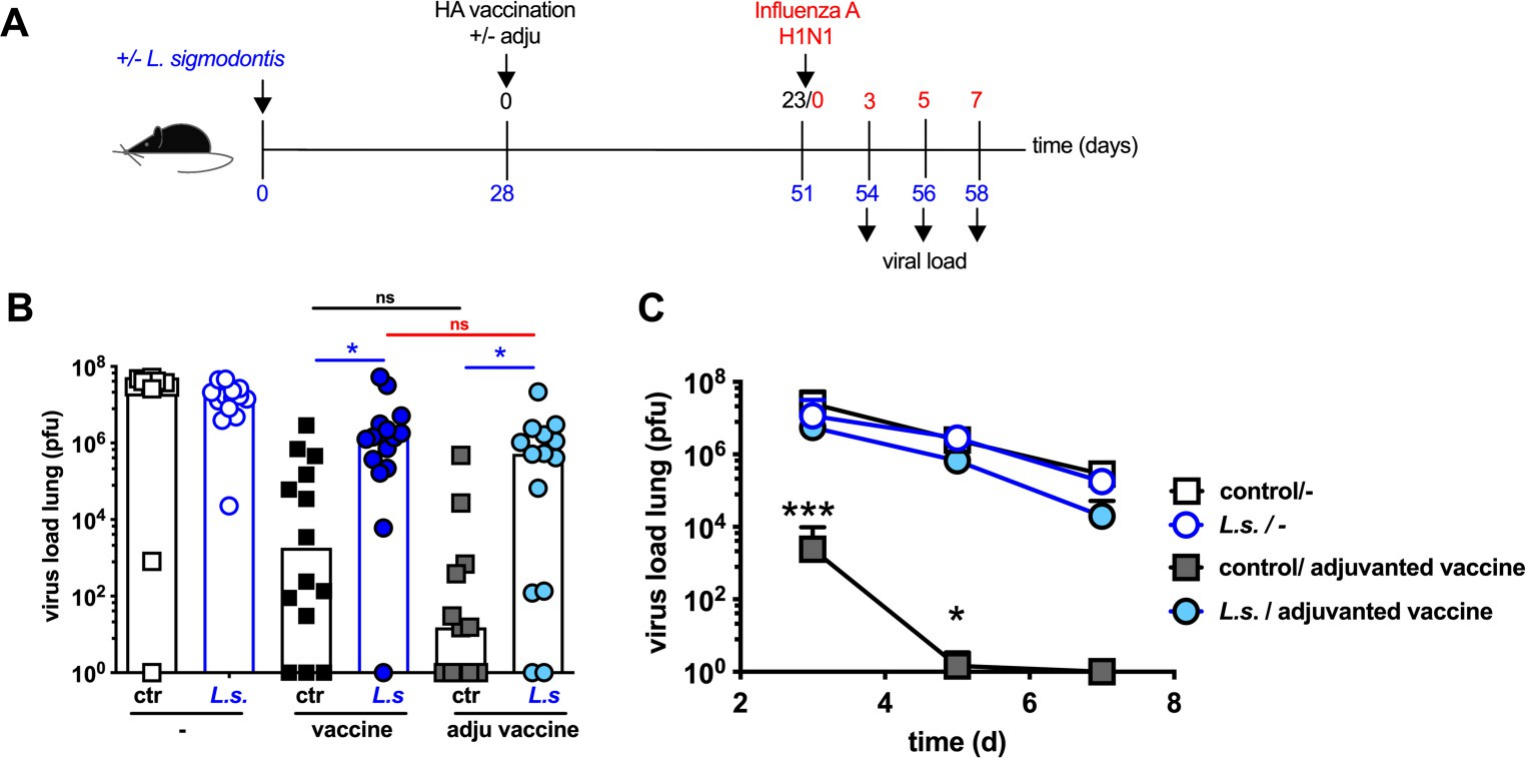
Adjuvanted influenza vaccination does not protect *L*. *sigmodontis*-infected mice from influenza virus replication in the lung. **(A)** Experimental design: C57BL/6 mice were left naive (black and grey squares) or naturally infected with *L*. *sigmodontis* (light and dark blue circles). Mice either received a control injection (open symbols) or were i.p. vaccinated with non-adjuvanted influenza vaccine Begripal season 2017/18 (black squares, dark blue circles) or adjuvanted influenza vaccine Fluad season 2017/18 (grey squares, light blue circles) at day 28 post *L*. *sigmodontis* infection. All mice were i.n. infected with 1 x 10^3^ PFU 2009 pH1N1 influenza A virus 2 days later. Influenza virus burden in the lung was quantified day 3 post challenge infection **(B)** or at indicated time points **(C)**. Shown are combined results out of 2 independent experiments with n ≥ 4 per group and experiment. **(B)** Each symbol represents an individual mouse the lines show the mean. Asterisks indicate statistically significant differences applying 1-way ANOVA (p > 0.001) with Tukey’s multiple comparison test comparing all vaccinated groups with each other. Numbers indicate p-values, ns = not significant, * *p* ≤ 0.05, ** *p* ≤ 0.01, *** *p* ≤ 0.001; black asterisks: naïve control non-adjuvanted vaccine group to naïve control adjuvanted vaccine group, blue asterisks: *L*. *sigmodontis*-infected non-adjuvanted and adjuvanted vaccine groups to naïve control non-adjuvanted or adjuvanted vaccine groups, red asterisks: *L*. *sigmodontis*-infected non-adjuvanted to *L*. *sigmodontis*-infected adjuvanted vaccinated groups. **(C)** Symbols show the mean per group at the indicated time point, error bars show SD. Asterisk indicated differences between the naïve control adjuvanted vaccine group and the *L*. *sigmodontis*-infected adjuvanted vaccine group applying 2-way ANOVA with Sidak`s multiple comparison test.

Data were analysed using Graph Pad Prism, testing for normality distribution and further tested either with 1-way ANOVA with Tukey’s multiple comparison test (parametric) or Kruskall-Wallis with Dunns multiple comparison test (non-parametric) were performed. For comparison of body weight changes or virus load over time between 2 groups the 2-way ANOVA with Sidak`s multiple comparison test was performed. Statistical tests are indicated in the Figure legends. Asterisks for all analyses * p ≤ 0.05, ** p ≤ 0.01, *** p ≤ 0.001, **** p ≤ 0.0001. Combined sample sizes per group were ≥ 18 for the experiment depicted in Fig 1B and 1C, ≥ 12 for Fig 1C, ≥ 16 for Fig 2B and 2C, ≥ 10 for Fig 2D, ≥ 8 for Fig 2E and Fig 3B and 3C.

## Results

### Non-adjuvanted prime-boost vaccination protects helminth-infected mice from influenza infection-induced weight loss

Investigating the interference of *L*. *sigmodontis* infection with vaccination efficacy, we found that both a concurrent and a past helminth infection impaired the Ab response of all isotypes to seasonal Influenza vaccine [23]. This resulted in diminished protection against a challenge infection with 2009 pH1N1. Here, we aimed at establishing vaccination regimens that confer protection against influenza infection in helminth-infected mice. As a first approach, repeated vaccination was performed. Although it is not usually done with seasonal influenza vaccinations in the adult human population, repeated vaccinations are a commonly used strategy to boost vaccination responses.

Mice were either naturally infected with *L*. *sigmodontis* by exposure to infected mites or left naïve (Fig 1A). Subsequently, mice were either vaccinated once day 14 or twice days 14 and 28 post *L*. *sigmodontis* infection using the influenza vaccine Begripal season 2017/18. Begripal is a non-adjuvanted trivalent split subunit vaccine that is composed of HA derived from three different influenza strains including 2009 pH1N1 influenza A virus. Vaccine-induced protection is predominantly mediated by neutralizing Ab [27]. Vaccination of naïve control mice induced HA-specific IgG1 (Fig 1B) and neutralizing Ab response recorded as HI titre (Fig 1C) in all groups that received either one (black and dark blue symbols) or two (grey and light blue symbols) injections of the vaccine while non-vaccinated mice did not contain HA-specific Ab (Fig 1B and 1C open symbols). Thereby, the prime-boost vaccination regimen yielded significantly higher Ab and HI titres than just one dose of vaccination (Fig 1B and 1C black asterisks). Concurrent infection with *L*. *sigmodontis* at the moment of vaccination significantly reduced Ab and HI titres (blue asterisk) compared to naïve control mice, as described before [23]. This helminth infection-induced reduction of Ab and HI titres was still observed in mice that received a boost immunization, merely at an elevated level. Thereby, the Ab and HI titres in helminth-infected mice that received prime-boost vaccinations were more than 10-fold higher than in helminth-infected mice that were just vaccinated once (Fig 1B and 1C red asterisks).

To test if this elevated Ab response was sufficient to confer protection, a challenge infection was performed. To this end, all groups including non-vaccinated control mice were i.n. infected with a sub-lethal dose of a patient isolate from 2009 pH1N1 influenza A virus [28] that replicates in mice without further adaptation [26, 29, 30]. As sublethal infection results in a transient weight loss reflecting pathology, body weight was recorded over 14 days (Fig 1D). While non-vaccinated mice lost up to 20% of their body weight before recovering, non-helminth-infected control mice that were vaccinated once or twice were almost completely protected from influenza infection-induced weight loss. Helminth-infected mice that received a single vaccination displayed significantly increased weight loss compared to vaccinated control mice (Fig 1D, dark blue circles and blue asterisks), as we had shown before [23]. Still, vaccination had some protective effect also in helminth-infected mice as non-vaccinated helminth-infected mice lost significantly more weight than the vaccinated helminth-infected group (Fig 1D open blue circles to dark blue circles and black asterisks). By contrast, helminth-infected mice that received two vaccinations (Fig 1D, light blue circles) were protected from challenge infection-induced weight loss to the same extent as non-helminth-infected control mice that received one vaccination (Fig 1D, black squares).

### Adjuvanted single vaccination protects helminth-infected mice from influenza infection-induced weight loss

Next, we tested the efficacy of adjuvanted vaccines to confer protection on the background of pre-existing helminth infection (Fig 2). To this end we compared the non-adjuvanted influenza vaccine Begripal to the seasonal 2017/18 vaccine Fluad that contained the same 2009 pH1N1 HA as Begripal but was additionally enhanced with MF59, an oil-in-water squalene-based adjuvant (Fig 2A). A single adjuvanted vaccination with Fluad resulted in 5-fold increased titres of HA-specific IgG1 and neutralizing Ab responses indicated by HI titres in both, helminth-free and helminth-infected mice (Fig 2B and 2C, black and red asterisks). While IgG1 responses were rescued in helminth-infected mice that received adjuvanted Fluad vaccination (Fig 2B, p = 0.60), the HI titre, indicative of neutralizing capacity, was still 10-fold reduced in adjuvanted Fluad-vaccinated helminth-infected mice compared to control mice (Fig 2B and 2C, light blue asterisks). Nevertheless, adjuvanted Fluad-vaccinated helminth-infected mice were protected from the transient weight loss induced by a sublethal influenza challenge infection (Fig 2D and 2E) while non-adjuvanted Begripal-vaccinated helminth-infected mice lost up to 10% of their body weight (Fig 2D and 2E blue asterisks). Still, a residual protection was achieved in helminth-infected mice by application of a single non-adjuvanted Begripal to helminth-infected mice, since non-vaccinated mice lost even more weight (Fig 2D and 2E, black and grey asterisks), as observed above (Fig 1D).

Taken together, these results suggest that basic vaccination regimen improvements such as either repeated vaccination or introduction of mild adjuvants elevate Ab response to a level that sufficiently restores vaccine-induced protection from influenza infection-induced pathology also on the background of pre-existing helminth infections.

### Adjuvanted single vaccination does not protect helminth-infected mice from influenza virus replication in the lung

As the ultimate goal of vaccination is the induction of sterile immunity, we next quantified virus burden in the lungs of vaccinated and challenge infected mice (Fig 3). Vaccination of naïve control mice with either non-adjuvanted Begripal or adjuvanted Fluad significantly reduced the influenza A virus burden at day 3 post challenge infection compared to non-vaccinated mice (Fig 3B). Non-adjuvanted Begripal-vaccinated mice that carried a chronic helminth infection at the moment of vaccination displayed significantly elevated influenza A virus burden compared to the naïve control group, as expected [23]. To our surprise, helminth-infected mice that received the adjuvanted Fluad vaccination displayed the same high virus load in the lungs as helminth-infected mice that received the non-adjuvanted Begripal vaccination (Fig 3B dark blue circles to light blue circles). That was despite the elevated Ab responses (Fig 2B and 2C) and diminished influenza A virus infection-induced weight loss (Fig 2D and 2E), that we recorded in helminth-infected mice receiving the adjuvanted Fluad vaccination.

It should be noted that although the vaccinated control mice displayed reduced virus burden in the lungs compared to non-vaccinated mice, no sterile immunity was established at the time point of analysis i.e. day 3 post influenza A virus challenge infection. Therefore, we asked if potential advantages of the adjuvanted Fluad vaccination would be more pronounced at later time points and performed a kinetic study to record viral clearance (Fig 3C). Non-helminth-infected control mice that received an adjuvanted vaccination with Fluad cleared the 2009 pH1N1 influenza A virus from the lung day 5 post challenge infection while helminth-infected mice failed to clear the virus from the lung, even at later time points. Thereby the virus burden in non-vaccinated mice, either with or without underlying helminth infection, was comparable to the virus burden in helminth-infected mice that received the adjuvanted Fluad vaccination.

In summary, these findings show that protection from pathology such as influenza virus infection-induced weight loss does not strictly correlate with accelerated clearance of virus from the lung. Although vaccinating helminth-infected mice with the adjuvanted influenza vaccine Fluad elevated Ab responses and prevented influenza virus challenge infection-induced weight loss, clearance of influenza virus from the lung was not achieved.

## Discussion

Accumulating evidence suggests that the immunomodulatory effect of helminth infections spills over to non-helminth antigens and thus may compromise vaccination efficacy [7, 12, 31]. Having shown previously that concurrent and past *L*. *sigmodontis-*infection interfered with influenza vaccination efficacy [23] we here aimed at introducing basic and easy-to-implement improvements of the vaccination regimen. We report that both, prime-boost vaccination with non-adjuvanted trivalent split subunit vaccine Begripal or single vaccination with the MF59-adjuvanted vaccine Fluad elevated IgG1 and neutralizing Ab responses to influenza vaccination in helminth-infected mice. However, neutralizing Ab responses were still significantly reduced in helminth-infected mice compared to naive control mice. The partially restored Ab response was sufficient to protect mice from influenza challenge infection-induced weight loss but not from viral replication in the lung. Thereby, performing a kinetic analysis, we showed that Fluad-vaccinated mice cleared 2009 pH1N1 influenza A virus from the lung by day 5 p.i. whereas mice that were Fluad-vaccinated while carrying a chronic helminth infection displayed the same high viral burden as non-vaccinated mice.

Regarding the mechanism of helminth-induced suppression of vaccination efficacy, we have previously identified expanding type 1 regulatory T cells (Tr1) as one mediator of suppressed Ab responses to vaccination [23] and immunization or CD4^+^ T helper cell activation [18, 20, 21]. In the current study we did not pursue the underlying immunological mechanisms but rather aimed at outcompeting the helminth-induced suppression by improved vaccination regimen.

The influenza vaccine Begripal is a subunit trivalent inactivated vaccine, composed out of HA derived from 3 different influenza strains including 2009 pH1N1. Vaccination-induced protection is thought to be mediated predominantly by HA-specific neutralizing Ab [27]. Begripal is recommended for humans over 6 months age, contains no further adjuvant and thus is not very immunogenic. Therefore, the use of adjuvanted vaccines such as Fluad is recommended for elderly persons above 65 years, suffering from immune senescence [32]. Likewise, improved influenza vaccination strategies are currently evaluated for individuals with a compromised immune system due to HIV infection or immunosuppressive treatment of autoimmune diseases or after solid organ transplantation [33]. However, no strategies are approved for helminth-infected individuals that display an impaired immune response to bystander antigens as well [3–5]. Fluad contains the adjuvant MF59 which is a squalene-based oil-in-water adjuvant. Although the exact molecular mechanisms are not fully understood [25], MF59 was shown to induce upregulation of a broad array of immune-relevant genes [34], to improve APC recruitment and function [35, 36] and to enhance Ab responses, especially in children [37]. While adults usually receive a single vaccination against seasonal influenza, young children benefited from a second vaccination dose that resulted in significantly improved protection from influenza disease [38]. In line with these findings, we observed increased Ab responses in mice receiving a prime-boost vaccination either twice with the non-adjuvanted Begripal or a single vaccination with the MF59-adjuvanted Fluad, thus demonstrating that helminth-infection did not interfere with boost or adjuvant functions per se. However, the pre-existing helminth infection still reduced the Ab response to vaccination, leading to only partial establishment of protection. From the translational point of view protection from morbidity indicated by prevention of the influenza-induced weight loss appears to be advantageous enough. Nevertheless, and in light of even more fatal viral diseases such as Ebola, sterile immunity i.e. prevention of viral replication would be the ultimate goal.

The fact that basic improvements of the vaccination regimen with split subunit vaccines such as Begripal and Fluad did not induce sterile immunity in *L*. *sigmodontis-* infected mice is in line with our previous work demonstrating the general susceptibility of protein antigens to helminth-induced bystander immunosuppression [18, 20, 23]. This may also be extended to other vaccines since similar susceptibility to helminth-induced suppression was reported for a protein-polysaccharide conjugate vaccine against *Streptococcus pneumoniae* infection in mice [39]. Chronic *Taenia crassiceps* infection reduced *S*. *pneumoniae-*specific Ab response even if the overall Ab response was boosted by a second immunization. The reduced Ab response in *T*. *crassiceps-*infected mice resulted in impaired protection against pneumonia and increased lethality during a *S*. *pneumoniae* challenge infection. Likewise, chronic infection with the intestinal parasite *Heligmosomoides polygyrus* reduced the Ab response to vaccination with a crude lysate of *Plasmodium chabaudi* parasitized erythrocytes, thereby abrogating the protection from parasitaemia that was achieved in vaccinated helminth-free mice [40].

Moreover, helminth-induced suppression of vaccination efficacy is not restricted to protein-based vaccines. The efficacy of the DNA-based transmission-blocking anti-*Plasmodium falciparum* vaccine Pfs25 was compromised in mice with concurrent *H*. *polygyrus* infection despite repeated immunizations [41]. Interestingly, in the same study the immunization with irradiated *Plasmodium yoelii* sporozoites induced comparable circumsporozoite (CSP)-specific Ab responses and protection from a challenge infection in *H*. *polygyrus*-infected mice, suggesting that life attenuated vaccines may outcompete helminth-induced immunosuppression. In line with this reasoning, we have shown previously that experimental immunization with a fusion protein composed out of the *Plasmodium berghei* CSP_245-253_ and detoxified Bordetella pertussis adenylate cyclase toxin (ACT) induced a CSP-specific CD8^+^ T cell response that conferred protection against *P*. *berghei* infection [42]. The numbers and the function of vaccination-induced CSP-specific T cells were reduced in mice that carried chronic *L*. *sigmodontis* infection during vaccination. Consequently, parasite burden in the liver was increased after a *P*. *berghei* sporozoite challenge infection. However, introduction of a prime immunization with a CSP-expressing attenuated live *Salmonella* strain before the ACT-CSP immunization rescued the CD8^+^ T cell response in *L*. *sigmodontis-*infected mice and conferred almost sterile immunity to a *P*. *berghei* challenge infection [22, 43]. The same advantage of life vaccine carrier was observed in anti-HIV immunization. Infection with the liver fluke *Schistosoma mansoni* reduced the cellular response to vaccination with a multi epitope T cell DNA vaccine against HIV-1 C [44]. By contrast, vaccinating with a HIV-1 gag protein expressing *Lysteria monocytogenes* as living carrier induced a gag-specific T cell response in *S*. *mansoni-*infected and non-infected mice to the same extent [45].

In light of our failed attempts to rescue the influenza vaccination-induced protection by prime-boost vaccination or mild adjuvants, these combined findings raise the concern that not all vaccine regimens may induce a protective immune response in helminth-infected individuals. As also drug-induced deworming did not restore responsiveness to influenza vaccination in mice, a combination of deworming and improved vaccination regimen might be needed [24]. The accumulated data from the mouse system suggest that especially life carrier-based vaccines might be more functional in helminth-endemic areas. Still, introduction of more potent adjuvants appears to be an option, since a recent study reported that recombinant Pfs230 conjugated to a carrier protein and adjuvanted with Alhydrogel induced comparable Ab titres hat displayed equal transmission inhibiting capacities in the absence and presence of intestinal *H*. *polygyrus* infection [46]. In summary our study highlights the importance to test vaccination responses for their ability to protect on the background of pre-existing helminth infection. Here, it will be extremely interesting to analyse the performance of the newly developed mRNA-based SARS-CoV-2 vaccines in helminth-endemic areas as well as in the mouse system.

## Supporting information

S1 File Prism file containing the numerical data used to generate Fig 1. (PZFX)

S2 File Prism file containing the numerical data used to generate Figs 2 and 3. (PZFX)
